# Role of Average Volume Assured Pressure Support Mode (AVAPS) in the Management of Acute Exacerbation of Chronic Obstructive Pulmonary Disease With Type 2 Respiratory Failure

**DOI:** 10.7759/cureus.32200

**Published:** 2022-12-05

**Authors:** Aditi Maheshwari, Jaikishan Khatri, Gunjan Soni, Nitish Saini

**Affiliations:** 1 Respiratory Medicine, Sardar Patel Medical College, Bikaner, IND; 2 Anesthesiology, Sardar Patel Medical College, Bikaner, IND

**Keywords:** non-invasive ventilation, pulmonary obstructive disease, airway disease, lungs, pulmonology, bipap, copd, avaps

## Abstract

Background

Non-invasive ventilation (NIV) is a well-established approach in the treatment of acute exacerbation of chronic obstructive pulmonary disease (COPD) with type 2 respiratory failure. Average volume-assured pressure support (AVAPS) mode integrates the characteristics of both volume and pressure-controlled modes of NIV. In bilevel positive airway pressure (BiPAP) mode, volume is the dependent variable, whereas in AVAPS mode, pressure is the dependent variable. In this study, we aimed to compare the role of AVAPS mode with BiPAP spontaneous/timed (S/T) mode for the management of patients with acute exacerbation of COPD with type 2 respiratory failure.

Methodology

A hospital-based comparative and analytical study was carried out on 100 patients with acute exacerbation of COPD with type 2 respiratory failure admitted to respiratory disease hospital, Sardar Patel Medical College, Bikaner (Rajasthan, India). Patients were randomly divided into two groups of 50 patients each. Group A patients were treated with AVAPS mode and group B patients with BiPAP (S/T) mode. Arterial blood gases, average duration of hospital stay, and need for invasive mechanical ventilation were compared between the two groups.

Results

There was a statistically significant difference in favor of group A in terms of improvement in pH and pCO_2_ as compared to group B at 6 h (pH, p=0.027; pCO_2_, p=0.012) and 24 h (pH, p=0.032; pCO_2_, p=0.013). The duration of hospital stay was found to be lower in group A (p=0.003). However, no significant difference was found in terms of need for invasive mechanical ventilation between both groups (p=0.338).

Conclusion

Application of AVAPS mode results in more rapid and steady improvement in patients of COPD as compared to BiPAP (S/T) mode. Thus, management through non-invasive ventilation AVAPS mode should be considered in patients with acute exacerbation of COPD with type 2 respiratory failure.

## Introduction

Patients with severe chronic obstructive pulmonary disease (COPD) are susceptible to exacerbations, which might lead to respiratory failure and hospitalization. The most common trigger for exacerbations is infection. In acute hypercapnic respiratory failure, there is decrease in the level of pH with increase in the level of PaCO_2_ (respiratory acidosis).

Non-invasive ventilation (NIV) has gained popularity in the management of acute as well as chronic respiratory failure [1]. Unlike invasive ventilation, which uses an endotracheal tube, non-invasive ventilation uses nasal masks, oronasal masks, full face masks, mouthpiece, or helmet to provide respiratory support [2]. Non-invasive ventilation achieves adequate gas exchange correcting hypoxemia and hypercapnia without causing complications of invasive ventilation. Currently, NIV is the preferred modality of treatment for exacerbation of chronic obstructive pulmonary disease with respiratory failure [3,4].

In bilevel positive airway pressure (BiPAP) ventilator, we set an inspiratory positive airway pressure (IPAP) and expiratory positive airway pressure (EPAP). Adjustments in the values of IPAP change the tidal volume delivered for a given breath. The difference between the levels of IPAP and EPAP dictates the pressure support which combines with respiratory rate to determine the patient’s ventilation [5]. The disadvantage with BiPAP (S/T) mode is that it depends on the patient’s level of consciousness, position, and underlying lung compliance to provide adequate tidal volume, if any of these factors change during a patient’s hospital stay, it can reduce the tidal volume and thus ventilation, which can lead to patient’s deterioration [6]. This problem can be resolved by the use of average volume assured pressure support (AVAPS) mode.

AVAPS mode of non-invasive ventilation integrates the characteristics of both volume and pressure-controlled modes of non-invasive ventilation. AVAPS mode provides the advantage of delivering a fixed tidal volume, which is kept constant due to changes in the levels of inspiratory pressures. The pressure support is not fixed as the AVAPS mode uses a range of values for the IPAP, a maximum and a minimum IPAP. IPAP changes on its own in synchronization with changes in the patient’s respiratory effort, lung compliance, or extrinsic lung resistance [7,8]. The ventilator attains this on the basis of feedback response which changes the inspiratory pressure from breath to breath to deliver the pre-set tidal volume. Changes in the levels of inspiratory pressure take place smoothly so that there is no patient ventilator asynchrony and discomfort [9].

The use of AVAPS mode in the management of acute hypercapnic respiratory failure has shown rapid clearance of carbon dioxide as compared to BiPAP (S/T) mode since it has the advantage of delivering a fixed tidal volume [10].

## Materials and methods

**Figure 1:**
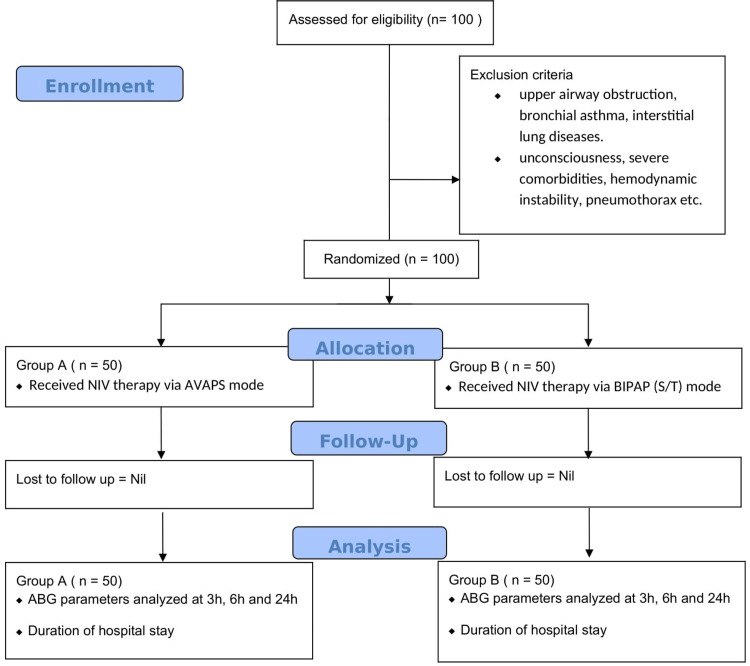
Flowchart of the study.

This hospital-based comparative and analytical study was conducted in the Department of Respiratory Medicine, Sardar Patel Medical College and A.G. Hospitals, Bikaner, Rajasthan, from January 2021 to December 2021. Approval from the institutional ethical committee and informed consent from the subjects or their legal relatives were obtained. One hundred patients with acute exacerbation of COPD with type 2 respiratory failure were included in the study using medical statistical software, which were then randomly categorized into two groups of 50 patients each. Patients in group A were given therapy with AVAPS mode and in group B, patients were treated with BiPAP (S/T) mode (Figure 1).

This study aimed to compare the role of AVAPS mode with BiPAP (S/T) mode in the management of acute exacerbation of chronic obstructive pulmonary disease patients with type 2 respiratory failure.

Primary outcome

The primary outcome of the study includes changes in ABG parameters such as pH, PaCO_2_, and PO_2_ after 3 h, 6 h, and 24 h of NIV application.

Secondary outcome

Comparison of Duration of Hospital Stay and Need for Invasive Mechanical Ventilation

Patients who were known cases of chronic obstructive pulmonary disease (on the basis of the clinical history, physical examination, spirometry, and chest film) with acute exacerbations with type 2 respiratory failure (PaCO_2_ >45 mm Hg, arterial pH <7.35, and respiratory rate >24 breaths/min), severe dyspnea with clinical signs suggesting of respiratory muscle fatigue, increased work of breathing, or both (such as use of respiratory accessory muscles, paradoxical motion of abdomen, or retraction of costal spaces), and persistent hypoxemia despite supplemental oxygen therapy were included in this study.

Exclusion criteria included unconsciousness, uncooperativeness, severe comorbidities, facial trauma, hemodynamic instability, ventricular or atrial arrhythmia, pneumothorax, aspiration, upper airway obstruction, bronchial asthma, interstitial lung diseases, and cardiopulmonary arrest.

After the recruitment, consent, and randomization into AVAPS and BiPAP (S/T) groups, patients were explained about the technique of the device and then started on NIV treatment given via Philips Trilogy EV300 ventilator (Amsterdam, Netherlands: Philips) through non-vented face mask according to individual basis with targeted oxygen saturation between 88% and 92%. Patients were assessed using arterial blood gas (ABG) parameters at 3 h, 6 h, and 24 h of NIV use. In addition to NIV treatment, both groups received the standard therapy, including corticosteroids, nebulized bronchodilators, and antibiotic therapy.

In AVAPS mode, initial settings were set with an IPAP maximum of 26 cm of H_2_O, IPAP minimum of 12 cm of H_2_O, and expiratory positive airway pressure (EPAP) of 6 cm of H_2_O. The target tidal volume (Vt) in our patients was set to 6-8 mL/kg of ideal body weight (IBW). Respiratory rate was kept at 18 breaths/min and inspiratory time was set at a minimum of 0.8 s. In BiPAP, S/T mode initial settings were set with an IPAP of 14 cm of H_2_O and EPAP of 6 cm of H_2_O. The respiratory rate was set at 18 breaths/min and inspiratory time was set at a minimum of 0.8 s. Ventilatory settings in both groups were adjusted according to the patient’s requirements.

ABG parameters were measured at 3 h, 6 h, and 24 h of NIV application and were compared in both groups. After 24 h, the criteria for intubation included cardiopulmonary arrest, respiratory pauses with loss of consciousness, worsening psychomotor agitation, worsening mental status, and hemodynamic instability. The average duration of hospital stay and need for invasive mechanical ventilation was also compared between the groups.

Statistical analysis

To collect required information from eligible patients, a pre-structured pre-tested proforma was used. Data were coded and recorded in MS Excel spreadsheet program. SPSS version 23 (Armonk, NY: IBM Corp.) was used for data analysis. Descriptive statistics were elaborated as means/standard deviations for continuous variables, and frequencies and percentages for categorical variables. Data were presented in a graphical manner wherever appropriate for data visualization. The mean values were compared using non-parametric tests Wilcoxon-Mann-Whitney U test or parametric Student's t-test. For categorical variables, χ2 or Fisher's exact tests were used. A value of p<0.05 was considered significant.

## Results

There was no statistically significant difference in the demographic profile of the patients. The two groups were comparable in regard to age and sex (p>0.05). The baseline ABG was comparable in both groups (Table 1).

**Table 1:** Association of different groups with demographic and initial baseline parameters. *Fisher’s exact test. **Chi-square test. ***Wilcoxon-Mann-Whitney test.

Variable		Group A	Group B	p-Value
Age (years)		60.32 (12.05)	57.50 (10.59)	0.209^*^
Gender	Male	36 (72.0%)	38 (76.0%)	0.648^**^
	Female	14 (28.0%)	12 (24.0%)	
Initial pH		7.23 (0.04)	7.24 (0.04)	0.173^***^
Initial PaCO_2_		87.74 (5.58)	88.22 (5.39)	0.772^***^
Initial PaO_2_		54.06 (7.10)	52.40 (4.73)	0.135^***^
Initial SaO_2_		72.00 (9.73)	68.28 (11.59)	0.322^***^

The progress of the patients was traced clinically and by repeated ABG parameters analysis after 3 h, 6 h, and at 24 h of NIV application. It was observed that ABG parameters improved in both groups at 3 h after NIV application; however, there was no statistically significant difference between both groups in terms of pH, PaCO_2_, and PaO_2_.

**Figure 2:**
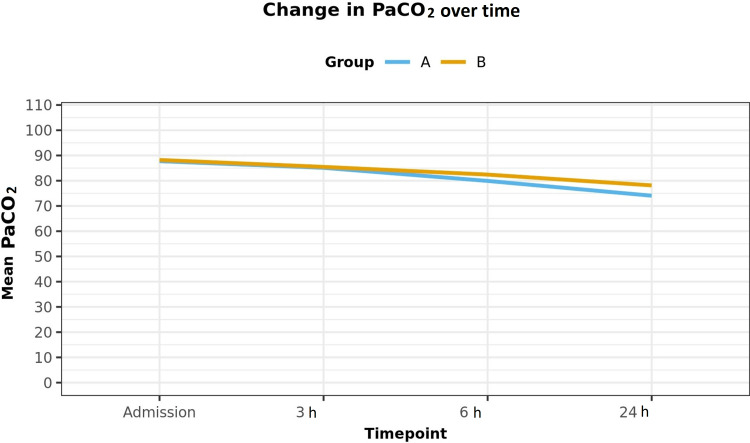
Change in PaCO2 over time.

**Figure 3:**
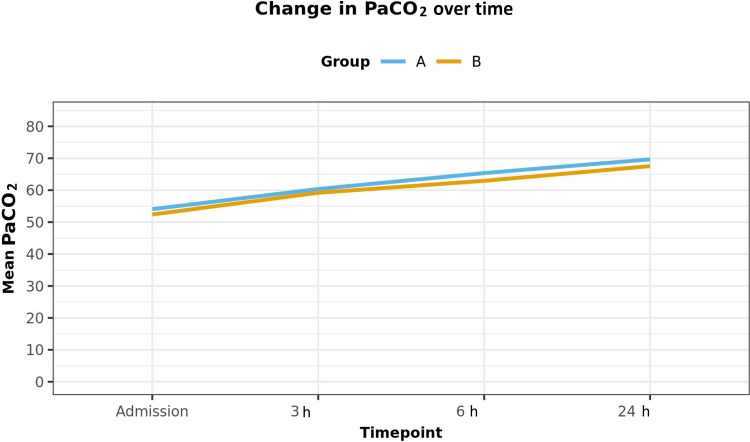
Change in PaO2 over time.

However, at 6 h and 24 h, there was a significant improvement in terms of pH (Table 2), PaCO_2_ (Figure 2), and PaO_2_ in both groups (Figure 3). But the improvements in pH, PaCO_2_ (p<0.001), and PaO_2_ (p<0.001) were much more significant in group A as compared to group B.

**Table 2:** Comparison of the two groups in terms of change in pH over time. SD: standard deviation

pH	Group		P-value for comparison of the two groups at each of the timepoints (Wilcoxon-Mann-Whitney test)
	A	B	
	Mean (SD)	Mean (SD)	
3h	7.25 (0.03)	7.26 (0.03)	0.290
6 h	7.30 (0.03)	7.28 (0.03)	0.027
24 h	7.33 (0.05)	7.31 (0.06)	0.032
Overall p-value for comparison of change in pH over time between the two groups (generalized estimating equations)	<0.001		-

There was a statistically significant difference between the two groups in terms of duration of hospital stay (p=0.003). No significant difference between the two groups in terms of need for invasive ventilation (p=0.338) was observed (Table 3).

**Table 3:** Duration of hospital stay and need for invasive mechanical ventilation (mean±SD). ^*^Wilcoxon-Mann-Whitney U test. ^**^Chi-squared test.

	Group A	Group B	p-Value
Duration of hospital stay (days)	8.54 (2.29)	9.90 (2.59)	0.003^*^
Need for invasive ventilation	4 (8.0%)	7 (14.0%)	0.338^**^

## Discussion

Our study showed that the use of AVAPS mode of NIV in patients with acute exacerbation of COPD with type 2 respiratory failure produces early improvement in parameters of arterial blood gases as compared to BIPAP (S/T) mode.

Hybrid modes of NIV have been developed like AVAPS mode which combines the benefit of both pressure-targeted and volume-targeted ventilation. NIV with AVAPS mode uses a fixed tidal volume that automatically adjusts to the patient's needs. As compared to BIPAP (S/T) mode, application of AVAPS mode is more comfortable and effective for patients, as the fixed IPAP in BIPAP mode is not able to deliver the required tidal volume that occurs as a result of dynamic changes in lung mechanics during exacerbation in COPD. Because of this advantage, AVAPS helped in the early washout of CO_2_ and thus resulting in a smaller number of days of hospital stay as compared to BIPAP (S/T) in our study.

When monitoring patients over 24 h, we observed a significant improvement in terms of pH, PaCO_2_, and PaO_2_ at 6 h and 24 h. Likewise, Claudett et al. observed a rapid and significant improvement in ABGs and consciousness (GCS) in both groups; however, patients treated with BiPAP S/T+AVAPS improved much faster compared with patients treated with the conventional strategy [11]. This improvement in the AVAPS group was probably due to the fact that, in these patients, IPAP quickly reached the level required for maintaining appropriate tidal volume, and thus correcting the hypoventilation with consequent improvements in alveolar ventilation.

In our study, there was a statistically significant difference between the two groups in terms of duration of hospital stay. The mean duration of hospital stay (days) was lower in group A (8.54 ±2.29) than in group B (9.90±2.59), p=0.003. Likewise, Ramchadaran et al. found significant difference in the two groups in terms of duration of hospital stay in favor of group 1 (BIPAP with AVAPS) as compared to group 2 (BIPAP S/T), p<0.001 [12]. Similarly, in a study done by Bajaj et al., it was observed that the length of stay in intensive care unit and hospital stay was less in the non-intubated as compared to intubated groups in patients with acute hypoxic respiratory failure [13].

Also, we found that there was no significant difference between the two groups in terms of need for invasive ventilation (χ2=0.919, p=0.338). Eight percent of the participants in group A required intubation while 14.0% of the participants in group B needed intubation. Similarly, Limsuwat et al. found no significant differences in BiPAP (S/T) with AVAPS compared with BiPAP (S/T) in terms of need for invasive ventilation [14].

Study limitations

Our study had some limitations. First was the small sample size. Another limitation is the short time of follow-up after starting NIV. We think a large randomized trial and longer follow-up period should have been done to test our hypothesis. Better outcomes would have been observed if the patients had been more compliant with NIV. Also, in some patients from both groups, significant air leak was present at the interface despite proper positioning of the mask which was also the reason for less improvement in ABG parameters than expected. Despite these limitations, we believe that this study provides valuable information, as it confirms the usefulness of AVAPS mode which ensures adequate inspiratory pressures and tidal volumes, facilitating a rapid correction of arterial blood gases.

## Conclusions

Both BIPAP and AVAPS are effective in the management of acute exacerbation of COPD with type 2 respiratory failure but application of AVAPS mode results into more rapid and steady improvement in clinical and ABG parameters as compared to BIPAP (S/T) mode as well as results in shorter duration of hospital stay. Thus, AVAPS should be considered as preferred mode of NIV for patients with acute exacerbation of COPD with type 2 respiratory failure.
